# Genome Sequence of an Ungulate Tetraparvovirus 1 from a Nasal Swab of an Irish Beef-Suckler Calf

**DOI:** 10.1128/mra.00911-22

**Published:** 2023-01-09

**Authors:** Kerrie Ní Dhufaigh, Matthew McCabe, Paul Cormican, Inmaculada Cuevas-Gomez, Mark McGee, Tara McDaneld, Bernadette Earley

**Affiliations:** a Teagasc, Animal & Grassland Research and Innovation Centre (AGRIC), Dunsany, County Meath, Ireland; b USDA, ARS, US Meat Animal Research Center, Clay Center, Nebraska, USA; Katholieke Universiteit Leuven

## Abstract

Here, we report the genome sequence of strain UTPV1/AB belonging to the species *Ungulate tetraparvovirus 1* (UTPV1). UTPV1/AB was isolated in the east of Ireland, directly from a nasal swab of a beef-suckler calf diagnosed with bovine respiratory disease on a farm in County Meath (longitude, 6°65′W; latitude, 53°52′N).

## ANNOUNCEMENT

Ungulate tetraparvovirus 1 (UTPV1), previously classified as *Bovine hokovirus* (1), is a small, linear, single-stranded DNA (ssDNA) virus that belongs to the genus *Tetraparvovirus*, in the family *Parvoviridae* (2). The virus has been identified from domestic yaks in China (3) and in cattle feedlots by metagenomic sequencing (4). Here, we report an UTPV1 genome sequence isolated from Ireland.

The UTPV1/AB strain was collected and sequenced directly from a nasal swab of a 6-month-old Aberdeen-Angus suckler calf (189 days old; weight, 318 kg) diagnosed with bovine respiratory disease (BRD) in November 2019 during a large-scale research study (5).

Prior to nucleic acid extraction, 1× phosphate-buffered saline (PBS) was added to the nasal swab and vortexed, 300 μL was removed, and then bead beating and RNase and DNase treatment steps were performed (6). Nucleic acids were extracted using the QIAamp MinElute virus spin kit (Qiagen, Manchester, UK) with carrier RNA substituted with 5 mg/mL linear acrylamide (ThermoFisher). Double-stranded cDNA was synthesized, using the Maxima H minus double-stranded (ds) cDNA kit (ThermoFisher) with random hexamers and purified using the GeneJet PCR purification kit (ThermoFisher). The rapid PCR barcoding kit (SQK-RPB004) from Oxford Nanopore and NEBNext Ultra II Q5 master mix (New England BioLabs Inc.) were used to generate Nanopore sequencing libraries. The library was loaded on a spot-on flow cell (FLO-MIN106D R9) (Oxford Nanopore Technologies, Oxford, UK) and sequenced on a MinION Mk1C sequencer with high-accuracy base calling.

Nanopore reads were filtered using Nanofilt (v2.8.0), and host-derived reads were removed resulting in 2,349 reads of viral origin with a mean length of 2,578 bp (7). *De novo* assembly was carried out using Flye (v2.9) (8). The Nanopore long reads were used to polish the Flye assembly using a single Medaka (v1.4.3) pass. The final draft consensus assembly was annotated using Prokka (v1.14.6) (9). The UTPV1/AB sequence contained 5,227 nucleotides. According to BLASTN (10) analysis, the near-complete genome sequence showed the highest nucleotide identity (99.53%) with UTPV1 strain HK-B38, a nonstructural protein from China (accession no. JF504698.1). UTPV1/AB had a much lower nucleotide similarity with any of the described ungulate tetraparvovirus genome sequences found in GenBank, with the highest similarity for Ungulate tetraparvovirus 1 isolate Yak hokovirus at 90.34% (accession no. KT225725.1).

**FIG 1:**
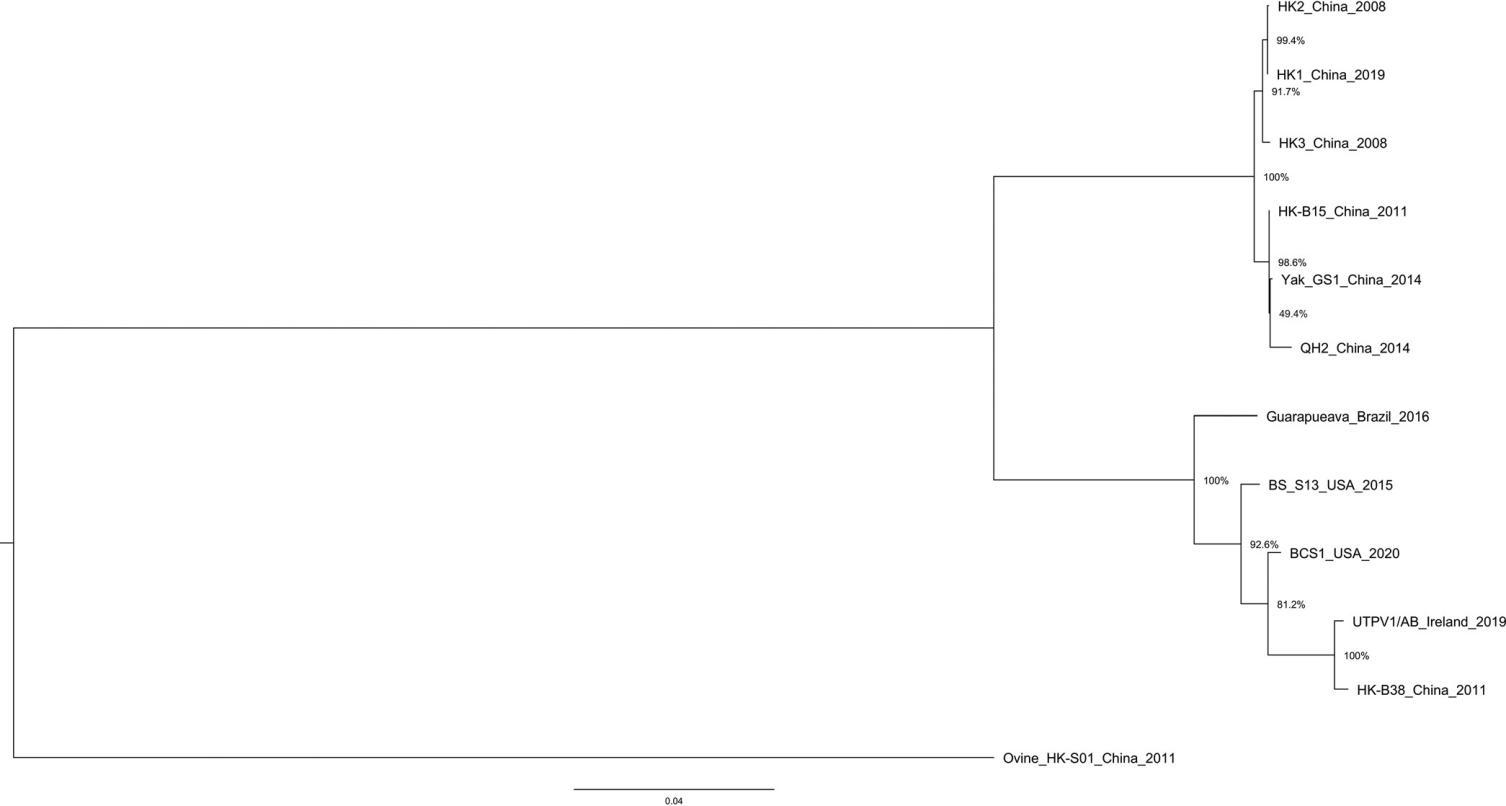
Phylogenetic tree of UTPV1/AB with Ovine HK-S01 as an outgroup to root the tree. Bootstrap values of >70% are displayed.

Multiple sequence alignment by the MUSCLE algorithm in MEGA11 (11, 12) of UTPV1/AB and 11 available UTPV1 genomes from GenBank was performed to infer phylogeny (Fig. 1). The phylogenetic analysis confirms that UTPV1/AB is closely related to UTPV1 strain HK-B38 and another UTPV1 isolate complete genome (accession no. MH814981.1) from China was also closely related. This UTPV1 complete genome was aligned against UTPV1/AB and their shared nucleotide identity was 99.56%.

Two hypothetical proteins were identified from the analysis, and their amino acid sequences were searched against the nonreductant protein sequence database of BLASTP to determine their identity. Hypothetical protein 1 had the highest similarity, at 99.84%, to a UTPV1 nonstructural protein (accession no. ANN02913.1). Hypothetical protein 2 was most similar to a minor structural protein from UTPV1 with an amino acid identity of 99.89% (accession no. AEQ76794.1).

### Data availability.

The genome sequence of UTPV1/AB has been deposited in GenBank under the accession number OP113956 and in the Sequence Read Archive under accession number SRR21529965.
